# Can digital technology innovation contribute to firms’ market value?

**DOI:** 10.1371/journal.pone.0309993

**Published:** 2024-09-05

**Authors:** Lijun Ran, Qi Zhang, Xin Li

**Affiliations:** School of Mathematics and Statistics, Beijing Technology and Business University, Beijing, China; Liaoning University, CHINA

## Abstract

With the rapid development of digital technology, digital technology innovation has become a core driver of China’s economic development. Thus, this study uses A-share listed companies from 2003 to 2021 as the research sample. The digital patents of firms are identified to portray the level of digital technology innovation by matching the digital economy industry classification code, national economy industry classification code, and IPC number. Considers the economic effect of digital technology innovation from the perspective of firm market value. It is found that digital technology innovation significantly contributes to the increase in firm market value, and this finding still holds when robustness tests are performed. Mechanistic tests have shown that digital technology innovation affects firm market value by driving digital transformation, promoting productivity, and enhancing market profitability. Further analysis reveals that digital technology innovation has a more significant effect on increasing firm market value for large, non-state, capital-intensive, technology-intensive and low internal control costs firms. This study verifies the enabling effect of digital technology innovation on the development of the real economy at the micro level, and provides insights for the optimization of China’s digital technology innovation policies and the formulation of firms’ digital development strategies.

## 1 Introduction

China’s economy has entered a stage of high-quality development, and is accelerating the construction of a new development pattern with the domestic general circulation as the main focus and the domestic and international dual circulation promoting each other. Higher requirements have been put forward for accelerating the development of the digital economy and promoting the deep integration of the digital economy and the real economy. Digital technological innovation, as the primary driving force for the development of China’s digital economy, is not only an important pathway for traditional firms to achieve digital transformation and empower their own high-quality development, but also a key to enhancing the competitiveness and influence of China’s digital economy (Tian et al., 2022) [1]. As the micro foundation of economic operations, firms serve as the key actors in digital technology innovation (Qi et al.,2021) [2]. The application of digital technology intensifies the competition among firms in terms of value provision. Therefore, the survival and development of firms inevitably face the fundamental issue of market competition. Then, can digital technology innovation enhance firm market value? What are the intrinsic mechanisms? With an increasing number of firms engaging in digital technology research and development innovation, the academic community urgently needs to explore the impact and mechanisms of digital technology innovation on firm development. This will provide insights for China to seize new opportunities in digitalization and expand new economic development spaces.

The impact of digital transformation on firm productivity has been analyzed in the literature from the perspectives of information asymmetry (Svahn et al.,2017) [3], production factor allocation (Acemoglu et al.,2018) [4], firm specialization division of labor (Yuan et al.,2021) [5], production and operation processes (Ciarli et al., 2021) [6], as well as from the perspectives of information technology (Bharadwaj et al. 1999) [7], big data (Bajari et al. 2019) [8], and other digital technology applications have explored the impact of digital technology applications on firm market value, while there is a gap in the research on digital technology innovation. Only a small amount of literature has discussed the impact of digital technology innovation on firm performance (Cathles et al., 2020; Liu et al., 2023) [9, 10]. In the face of the high threshold, high cost, and imitability of digital technology (Firk et al.,2021) [11], how to obtain economic benefits from digital technology innovation has become an important issue that firms need to think deeply about (Teece,2018) [12]. So it is necessary to link digital technology innovation to firm market value. Firm market value compared with indicators such as productivity and return on assets, contains both the value assessment of the current stage of firm development and reasonable expectations of future growth potential (Zhang et al., 2021) [13] Furthermore, the degree of digital technology innovation is not disclosed in firms’ annual reports and financial statements, leading to some difficulties in measuring it. Existing literature uses annual report keywords to measure the degree of digital transformation, which is conceptually distinct from digital technology innovation (Wu et al., 2021) [14], with the former emphasizing the application of digital technology and the latter emphasizing new breakthroughs in digital technology itself. Zhang et al. (2022) [15] used industry-specific patents, Liu et al. (2023) [10] used patent text analysis and Cathles et al. (2020) [9] used survey data to measure digital technology innovation. But there is no unified method for measuring digital technology innovation yet, thus it may cause the problem of over- or under-identification of digital technology innovation. Therefore, adopting Tobin’s Q to measure firm market value and explore the impact of digital technology innovation on firm market value, not only to provide evidence to support the theoretical mechanism of digital technology-enabled real economy development, but also to be able to supplement the relevant literature on digital economy and firm market value.

The main contributions are reflected in three aspects:(1) digital technology innovation is the frontier of academic research. Previous research in this area has been dominated by theoretical derivation (Acemoglu et al., 2019) [4], case qualitative(Svahn et al. 2017) [3], literature review(Kohli et al., 2019) [16], and narrative analysis (Tumbas et al., 2018) [17], with little empirical analysis. This study empirically researched and found that digital technology innovation has a significant effect on the market value of firms. It enriches the research on the economic effect of digital technology innovation of firms and provides theoretical support for digital technology to help the development of real economy.(2) Digital technology innovation is subdivided into digital technology invention patents and digital technology utility model patents. The research found that digital technology invention patents can significantly enhance the market value of firms, while digital technology utility model patents do not have a significant effect on the enhancement of market value of firms. It expands research on digital technology innovation for firm market value.(3) The mechanisms by which digital technological innovations enhance the market value of firms are explored. It was found that digital transformation, productivity and market profitability enhancement are important channels through which digital technology innovations increase the market value of firms. This provides insights to guide firms to improve productivity, enhance market profitability, and accelerate digital transformation.

## 2 Theoretical analysis

### 2.1 Digital technology innovation and firm market value

Digital technology innovation is the process and result of companies creating new products, processes, organizations, and business models based on digital technology (Yoo et al., 2012) [18]. As the innovation field that brings together the most innovative elements and has the widest prospect of development and application, digital technology innovation has a profound impact on all kinds of current socio-economic activities (Qu, 2022) [19]. Compared with other technologies, digital technology innovation will have a deeper impact on enterprise development and strongly enhance firm market value.

First, emerging digital technologies are embedded in all types of production and operation activities of physical firms, which can replace human labor in the production process, optimize the allocation of traditional production factors, reduce the dependence of firms on resource factors, change the original value creation model (Acemoglu et al., 2018) [4], thus promoting the transformation of firms’ business models, thus enhancing their market value. Second, traditional innovation has a long cycle from R&D to marketization, the final product does not fully meet the market demand, faces the risk of being eliminated in the continuously changing market demand. The self-growing nature of digital technology innovation by adding digital resources as a factor of production enables rapid upgrading and iteration of digital products and services (Tee et al.,2009) [20], improving customer satisfaction and promoting customer loyalty (Balci, 2021) [21], meeting the market’s high demand for digital intelligent products (Yoo et al., 2010) [22], contributing to increase the market value of the firm. Again, process innovation based on digital technology can change the traditional business processes of product development, manufacturing, purchasing and sales (Lyytinen et al., 2016) [23], which can not only improve production efficiency, but also better meet the personalized, diversified and multi-level consumer demand in the digital age. Digital technology can aggregate demand and supply information (Wei et al., 2022) [24]. Flexibing adjust its own production capacity according to the dynamic changes on the supply side and market side, realize the transformation of production capacity and supply chain management mode from traditional industrial mode to intelligent management mode, which in turn is conducive to enhancing firm market value (Huang et al.,2019) [25]. The application of digital technology can promote firms to obtain various data and information generated in the production management process more systematically and precisely. Breaking the path dependence in the traditional process management process through data computing and processing (Xiao,2020) [26]. Reducing the cost of information exchange and communication between firms and clients and the supply side (Brynjolfsson et al.,2011) [27], it helps to enhance firm market value. Finally, digital technology innovation can also break organizational or industrial boundaries (Nambisan et al., 2017) [28], change the value creation and acquisition mode of firms. Improve the connectivity among innovation participants, the efficiency of resource use, and the transparency of the innovation process through the sharing of technology and knowledge and the effective use of digital components, promoting the change of firm value acquisition, expanding the firm value space. Therefore, hypothesis 1 is proposed.

H1: Digital technology innovation have a promoting effect on firm market value.

### 2.2 Digital technology innovation, digital transformation and firm market value

Digital technology is the technological support for digital transformation of enterprises and its innovation process plays an important role in facilitating digital transformation (Bharadwaj et al., 2013) [29]. Digital technology innovation is conducive to the establishment of digital technology systems and digital production processes, which in turn enhance firm market value. The application of digital technologies such as big data can not only strengthen the processing capability of firms’ massive data (Zhang et al., 2021) [13], but through the collection and analysis of data at all macro and micro levels, firms can make more accurate market forecasts to improve decision-making efficiency (Brynjolfsson et al., 2011) [27]. The application of digital technologies enables the transformation of the production process of traditional industries into automation. Reduce the level of human involvement in the production and operation process, reduce the labor cost per unit of output (Brynjolfsson et al., 2017; Babina et al., 2021) [30, 31]. Thus, the value creation model of the firm is optimized and the value creation path of the firm is broadened (Grover et al., 2012; Henfridsson et al., 2018) [32, 33]. Digital technology innovation facilitates firms to establish digital product systems and digital business models to enhance firm market value. The introduction of new digital products and digital business models drives deep changes in the core market business of firms (Wu et al., 2021) [14]. Firms are able to break industry boundaries (Nambisan et al., 2017) [28] and continuously innovate the convergence of digital scenarios, which in turn leads to new growth poles of digital products and businesses and enhances firm market value. In addition, through the sharing of technology and knowledge and the effective use of digital components, digital technology innovation can overcome spatial and technological constraints (Yu et al., 2017) [34], improve the connectivity among innovation participants and the efficiency of resource use, can build an ecological value creation model and obtain richer market value. So digital technology innovation can facilitate the digital transformation of firms. Therefore, hypothesis 2 is proposed.

H2: Digital technology innovation can increase firm market value by facilitating digital transformation.

### 2.3 Digital technology innovation, production efficiency and firm market value

First, digital innovation drives the efficiency of information transfer within the firm (Yoo et al.,2012) [18], which in turn improves the firm’s ability to integrate resources (Svahn et al., 2017) [3]. Optimal allocation of resources is achieved by reallocating available resources to the most needed departments, increases operational efficiency and improves the financial performance of the firm (Liu et al., 2023) [10], enhancing firm market value. Second, digital innovation has penetrated into the design, management, and analysis processes of modern business operations and has the potential to improve operational efficiency (Mendling et al., 2020) [35]. Based on the data obtained by digital technology, companies can improve their production processes and adjust their production plans in a timely manner, which in turn can enhance their R&D innovation and operation management capabilities, increase their market value (Zhang et al., 2021) [13]. Again, digital technology can reduce the involvement of people in the production and operation process, reduce the room for manipulation of business processes, reduce the cost of external supervision, reduce the cost of internal control and improve productivity (Chen et al., 2022) [36]. Digital technology innovation promotes real-time and transparent key activities such as production processes, R&D processes, and financial control, thus reducing the cost of supervision in various business processes, reducing the efficiency loss caused by agency problems (Yuan et al., 2021) [5], improving productivity, thus increasing the firm market value. Therefore, hypothesis 3 is proposed.

H3: Digital technology innovation can increase firm market value by improving production efficiency.

### 2.4 Digital technology innovation, market profitability and firm market value

Innovation theory holds that innovation is the source of economic development and the decisive factor for firms to maintain their core competitiveness. Increased capital investment in R&D and innovation by firms can significantly improve firm performance (Li et al., 2020) [37]. Digital technology innovation can enhance the profitability of a firm in the product market, which is reflected in the capital market as an increase in the market value of the firm. Firstly, digital technology innovations have disrupted traditional business models and spawned new growth points for firm value creation. Digital technologies enhance, expand, and replace traditional business models through automation and digital augmentation, digital scaling, and digital transformation (Li,2020) [38]. Shifting from a closed to an open business model enables firms to flexibly respond to environmental changes, seize external opportunities, and improve their performance (Li et al., 2021) [39]. Secondly, digital technology innovation helps firms to fill the gap in the digital consumer market, creating a larger market revenue space for firms to enhance their market value. Relative to traditional products, the intangible part of digital technological innovation results constitutes most of the value of the new product (Liu et al., 2020) [40], while the firm’s innovation costs mainly come from the idea generation, research and development, and market research phases, with the implementation and dissemination phases costing much less, which undoubtedly has a greater return to scale (Guellec et al., 2017) [41], which, coupled with the existence of a market demand gap, will surely increase the firm’s market share and hence its market value. Finally, digital technology innovations have significant creative destruction potential, which leads to higher market value and capital return risk compensation for firms. Digital technological innovations are more market-disruptive than other technologies, resulting in more industries being affected by "winner-take-all" (Guellec et al., 2017) [41]. Digital technology innovations increase the risk of creative destruction in the marketplace by lowering the cost of innovation and reducing barriers to entry and exit in the digital product marketplace. In traditional markets, new products may reduce the market share of incumbent firms, while in markets of creative destruction, new products may directly result in incumbent firms losing market share altogether (Guellec et al., 2017) [41]. Therefore, the stronger the firm’s digital technology innovation capability, the higher the market risk compensation it receives, and the higher market returns significantly increase the firm’s market value. Therefore, hypothesis 4 is proposed.

H4: Digital technology innovation can increase firm market value by promoting market profitability.

## 3 Empirical study design

### 3.1 Data

Based on the availability and completeness of data, A-share listed companies from 2003–2021 are selected as the initial research sample. Patent data is obtained from PRC State Intellectual Property Office. Data of listed companies is obtained from CSMAR. Meanwhile, to ensure the validity and stability of the sample, the samples were processed as follow: (1) exclude the sample of firms in the ST category. (2) Exclude firms in the financial industry.(3) Exclude firms with wrong records and missing main indicators.(4) Exclude firms whose total number of digital technology patent applications is less than one. (5) Exclude the sample of firms whose total assets are less than total liabilities. This resulted in 12246 firm-year observations. In order to mitigate the effect of extreme values, the continuous variables are subjected to a top and bottom 1% tailing process.

### 3.2 Variable definition

Core explanatory variables. This article draws on Tao Feng et al. (2023) [42] to measure digital technological innovation. The main idea is to identify digital technology innovation patents that match the technical characteristics of digital innovation activities at the IPC level, with the help of technical information on patented innovation activities provided by the International Patent Classification (IPC). Specifically, firstly, combining the "Statistical Classification of the Digital Economy and Its Core Industries (2021)" published by the National Bureau of Statistics and the "Table of Reference Relationships between the International Patent Classification and the National Economic Industry Classification (2018)", constructing the "Digital Economy Core Industry Classification Code—Four-digit Code of the National Economic Industry Classification (SIC4)—International Patent Classification Panel (IPC)" correspondence, identifying the technical field to which digital technology innovation belongs and its corresponding IPC code, so as to identify the digital technology innovation patents filed by firms at the IPC panel level. Secondly, the identified digital technology invention patents and utility model patents are summed up from two dimensions of "firm-year" to construct a firm-level digital technology innovation measure (*LnDigiInno*).

Explained variables. The explanatory variable of interest in this paper, firm market value, is an important indicator of a firm’s ability to grow (Bharadwaj et al., 1999) [29]. Compared to financial performance indicators such as return on assets, Tobin’s Q is more sensitive to market response and better reflects the economic performance of firms (Luo et al., 2023) [43]. Tobin’s Q value contains not only the capital market’s value assessment of the firm’s current development status, but also the reasonable expectation of the firm’s future growth potential (Zhang et al., 2021) [13], which is more in line with the theme researched in this research. Therefore, selecting Tobin’s Q as a proxy variable for the market value of the firm, which is calculated as (total market value + total liabilities)/total assets.

Control variables. A series of control variables are selected (Huang et al. 2023; Tao et al. 2023; Zhang et al. 2021) [13, 42, 44]. Size, measured by the natural logarithm of total assets(*Size*). Roa, measured by the ratio of net profit to total assets(*Roa*). Duality, measured by whether the chairman and the managing director are both directors(*Duality*). Dirratio, measured by the number of independent directors as a proportion of the total number of directors(*Dirratio*). The percentage of the first largest shareholder (*OwnCon*1), measured by the number of shares held by the first largest shareholder as a proportion of the total number of shares, and the percentage of the second largest shareholder to the tenth largest shareholder (*OwnCon*2_20), measured by the number of shares held by the second largest shareholder to the tenth largest shareholder combined as a proportion of the total number of shares. Firm age (*LnAge*), the current year minus the listed year plus one, then take the natural logarithm. Fixed asset ratio (*PPE*_*TA*), measured as fixed assets divided by total assets. Gearing ratio (*Lev*), measured as the ratio of total liabilities to total assets. Firm leverage (*LevC*), total assets divided by shareholders’ equity. The regression analysis also controls for firm individual effects, time effects, and industry effects.

### 3.3. Model construction

To investigate the impact of digital technology innovation on the market value of firms, the benchmark estimation model was set as:

$${TobinQ}_{it}=\beta_{0}+\beta_{1}{DigiInno}_{it}+\beta_{3}{Control}_{it}+\gamma_{i}+\mu_{t}+\theta_{j}+\vartheta_{jt}+\varepsilon_{it}$$ (1)

Where, *i* denotes the firm, *t* denotes the year, and *j* denotes the industry. *TobinQ*_*it*_ is the market value of firm *i* in year *t*; *DigiInno*_*it*_ is the digital technology innovation level of firm *i* in year *t*; *Control*_*it*_ is the set of control variables; *γ*_*i*_ denotes individual fixed effect; *μ*_*t*_ denotes time fixed effect; *θ*_*j*_ denotes industry fixed effect; *ϑ*_*jt*_ denotes industry-years fixed effect; *ε*_*it*_ denotes the random perturbation term, and the standard errors are clustered at the province level.

Descriptive statistics as shown in Table 1. The mean value of the degree of digital technology innovation indicator (*LnDigiInno*) is 2.033 with a standard deviation of 1.227, indicating that there is a wide variation in the degree of digital technology innovation among the firms in the sample. The mean value of *TobinQ* is 2.061, which is generally consistent with the statistics of other listed firms in the literature (Wu et al., 2016) [45].

**Table 1:** Descriptive statistics.

Variables	N	mean	sd	min	max
TobinQ	12246	2.061	1.229	0.866	7.820
LnDigiInno	12246	2.033	1.227	0.693	5.964
Size	12246	22.21	1.337	20.01	26.52
Roa	12246	0.0398	0.0578	-0.223	0.191
Duality	12246	0.295	0.456	0	1
Dirratio	12246	0.383	0.0728	0.250	0.600
OwnCon1	12246	34.23	15.02	8.260	74.60
OwnCon2_10	12246	24.14	12.77	2.130	55.10
LnAge	12246	1.975	0.725	0.693	3.258
PPE_TA	12246	0.206	0.145	0.00782	0.658
LevC	12246	2.170	1.261	1.070	8.927
Lev	12246	0.419	0.193	0.0610	0.859

## 4 Empirical results and analysis

### 4.1 Benchmark regression test

Benchmark regression are shown in Table 2. Column (1) does not include control variables. The coefficient of *LnDigiInno* is positive at the 10% significance level, which indicates that firm digital technology innovation can significantly enhance firm market value. Column (2) adds control variables. The coefficient of *LnDigiInno* is positive at the 1% significance level, indicating that firms’ digital technological innovations can significantly increase firms’ market value. Columns (3)-(5) show that the coefficients of *LnDigiInno* are all significantly positive. This indicate that digital technology innovation has a significant positive impact on firms’ market value, and Hypothesis 1 is verified, which is consistent with the findings of Tao et al. (2023) [42].

**Table 2:** Benchmark regression test.

Variables	(1)	(2)	(3)	(4)	(5)
	TobinQ	TobinQ	TobinQ	TobinQ	TobinQ
LnDigiInno	0.023*	0.060***	0.030**	0.024**	0.018**
	(1.86)	(5.16)	(2.41)	(2.14)	(2.06)
Size		-0.336***	-0.389***	-0.441***	-0.591***
		(-15.52)	(-15.52)	(-19.84)	(-9.90)
Roa		4.571***	4.213***	3.071***	3.495***
		(15.27)	(14.83)	(17.72)	(13.38)
Duality		0.014	-0.018	-0.059**	-0.074**
		(0.46)	(-0.65)	(-2.13)	(-2.66)
Dirratio		0.853***	0.291**	0.157	0.301
		(5.62)	(2.18)	(1.26)	(1.53)
OwnCon1		-0.009***	-0.003**	-0.002	-0.000
		(-6.33)	(-1.98)	(-1.54)	(-0.01)
OwnCon2_10		-0.002	0.002	0.006***	0.009***
		(-1.44)	(1.58)	(4.17)	(6.42)
Lnage		0.381***	0.321***	0.604***	0.914***
		(12.21)	(10.63)	(13.54)	(15.17)
PPE_TA		-0.479***	-0.159	-0.025	0.087
		(-4.45)	(-1.37)	(-0.20)	(0.60)
LevC		0.044***	0.022	0.011	0.013
		(2.79)	(1.47)	(0.70)	(0.84)
Lev		-0.402***	0.018	0.359***	0.299
		(-2.96)	(0.13)	(2.81)	(1.58)
cons	2.063***	8.715***	9.430***	10.215***	12.702***
	(72.00)	(20.96)	(19.03)	(21.05)	(9.49)
Year	NO	NO	YES	YES	YES
Firm	NO	NO	NO	YES	YES
Industry	NO	NO	YES	YES	YES
Firm×Year	NO	NO	NO	YES	NO
Industry×Year	NO	NO	NO	NO	YES
*N*	12246	12246	12246	11616	11380
adj. *R*^2^	0.0096	0.0800	0.3164	0.6257	0.652

Notes: * p < 0.1

** p < 0.05

*** p < 0.01.

Do different types of digital technology innovation have different degrees of impact on firm market value? To subdivide digital technology innovation into digital technology invention patents(*LnDigiInnoA*) and digital technology utility model patents(*LnDigiInnoB*). Examine the impact of digital technology innovations from different types of digital technology innovations on firm market value. Column (1) of Table 3 shows that the coefficient of *LnDigiInnoA* is significantly positive at the 10% significance level, indicates that invention patents in digital technological innovation have a significant contributing effect on firm value. The coefficient of *LnDigiInnoB* in column (2) is positive but not significant. This indicate that the contribution of digital technological innovations to the market value of firms is mainly due to the impact of patents on digital technology inventions.

**Table 3:** Sub-Innovation types.

Variables	(1)	(2)
	TobinQ	TobinQ
LnDigiInnoA	0.013*	
	(1.72)	
LnDigiInnoB		0.010
		(0.97)
cons	12.693***	12.654***
	(9.49)	(9.45)
Control	YES	YES
Fixed effects	YES	YES
*N*	11380	11380
adj. *R*^2^	0.652	0.652

### 4.2 Robustness tests

Digital technological innovation (*L*.*LnDigiInno*) lags one period. Considering the limited impact of firms’ digital technological innovations on firms’ market value in the current period and the time-lagged nature of their enhancement of firms’ market value, the robustness estimation is re-estimated using the lagged one period of the digital technological innovation variable. The results of the robustness tests are shown in column (1) of Table 4, where the coefficient of *L*.*LnDigiInno* is positive at the 1% significance level, consistent with the benchmark results.

**Table 4:** Robustness test.

Variables	(1)	(2)	(3)
	TobinQ	Reduced sample	PSM-DID
L.LnDigiInno	0.069***		
	(6.51)		
LnDigiInno		0.029**	
		(2.48)	
Treat × Post			0.082***
			(3.07)
cons	9.184***	11.864***	9.329***
	(16.09)	(7.23)	(18.65)
Control	YES	YES	YES
Fixed effects	YES	YES	YES
*N*	7998	9462	24689
adj. *R*^2^	0.400	0.661	0.381

Shorter sample intervals(Reduced sample). Differences in the sample period may also have an impact on the estimation results, reducing the time interval of the sample to 2012–2021, thus examines the performance of the impact of firms’ digital technological innovations on market value in the time series of recent years. The results of the robustness tests are shown in column (2) of Table 4, where the coefficient of *LnDigiInno* is positive at the 5% significance level, consistent with the benchmark results.

PSM-DID. Benchmark regression results may suffer from endogeneity problems due to reverse causation. Therefore, PSM-DID was used for regression. First, calculates the propensity score of each sample firms using a logit model based on the firm characteristics measured by each control variable. Firms with at least one digital patent application are taken as the treatment group sample, the rest of the firms are taken as the control group sample. Using the one-to-one no-returns method and a standardized caliber of 0.1, all samples of treatment and control group firms during the sample period were extracted and matched to the control group sample with the closest propensity score in the same year.

Second, difference-in-differences is constructed as model (2). *Treat* is a dummy variable indicating the sample of the treatment group, takes a value of 1. *Post* takes the value of 1 after the firm’s first digital technological innovation, and 0 otherwise.


$${TobinQ}_{it}=\alpha_{0}+\alpha_{1}{Treat}_{t}\times {Post}_{i}+{Control}_{it}+\gamma_{i}+\mu_{t}+\theta_{j}+\vartheta_{jt}+\varepsilon_{it}$$ (2)

Column (3) of Table 4 shows: the regression coefficients of the cross-multiplier *Treat*×*Post* estimated by model (2) are significantly positive at the 1% significance level, indicating that the market value of the firms is significantly increased after the digital technology innovation. Benchmark regression results are robust.

### 4.3 Mechanism test

The previous theoretical hypothesis section suggests that digital technological innovations can affect firm market value through digital transformation, productivity and market profitability is digital technological innovations. According to Jiang (2022) [46], the identification suggestion regarding mechanism variables, empirical analysis should only focus on examining the impact of digital technology innovation on mechanism variables. This approach helps to avoid biases in the original mediation effect model. Referring to the established literature (Li et al., 2021) [47], the mediating effect is modeled as follows:

$${Mechanism}_{it}=\varphi_{0}+\varphi_{1}{DigiInno}_{it}+\varphi_{3}{Control}_{it}+\gamma_{i}+\mu_{t}+\theta_{j}+\vartheta_{jt}+\varepsilon_{it}$$ (3)

*Mechanism*_*it*_ denotes the three mechanism variables of digital transformation, production efficiency and market profitability. The other variables are consistent with model (1).

#### 4.3.1 Mechanisms for testing productivity

In checking the effectiveness of the channels to increase productivity, drawing on previous literature (Liu et al., 2015; Liu et al., 2016) [48, 49], total factor productivity (*TFP*) is used to measure the productivity of firms and examine the impact of digital technological innovations on the productivity of firms. It as an explanatory variable using model (3) for regression, the results in column (1) of Table 5 show that the regression coefficient of *LnDigiInno* is positive at 5% significance level, indicating that digital technological innovation significantly improves its own productivity, which in turn enhances the market value of the firm.

**Table 5:** Mechanism tests.

Variables	(1)	(2)	(3)	(4)
	TFP_LP	lnDigital	Profits	Retained
LnDigiInno	0.022**	0.025***	0.062***	0.062**
	(2.23)	(2.99)	(2.98)	(2.62)
cons	-4.863***	-1.169*	-13.629***	-13.247***
	(-20.23)	(-1.91)	(-9.99)	(-9.35)
Control	YES	YES	YES	YES
Fixed effects	YES	YES	YES	YES
*N*	12036	11380	11380	11380
adj. *R*^2^	0.832	0.850	0.813	0.815

#### 4.3.2 Digital transformation

Referring to Wu Fei et al.’s (2021) [14] measure of firms’ digital transformation, an indicator of the overall level of digital transformation was constructed using the word frequency data of firms’ digital transformation from the CSMAR database. Column (2) of Table 5 shows that the regression coefficient of *LnDigiInno* is significantly positive at the 1% significance level, indicating that digital technology innovation significantly improves the digital transformation of firms, enhances the market value of firms.

#### 4.3.3 Market profitability

Digital technology innovation can enhance the profitability of firms in the product market, which in turn can be reflected in the capital market as a continuous increase in the market value of the firm. Market profitability and internal capital profitability are used to measure firms’ market profitability. Market profitability is measured using undistributed earnings per share from the CSMAR database. Internal capital profitability is measured using retained earnings per share. Column (3) of Table 5 examines the effect of digital technological innovation on market profitability. The regression coefficient of *LnDigiInno* is positive at the 1% significance level, indicating that digital technological innovation can effectively improve the market profitability of firms. Column (4) tests the effect of digital technology innovation on the profitability of internal capital. The regression coefficient of *LnDigiInno* is positive at the 5% significance level, which indicates that digital technology innovation can effectively improve the profitability of firms’ internal capital. Therefore, digital technology innovation can significantly improve the profitability of the firm’s market, enhances the firm’s market value.

### 4.4 Heterogeneity test

#### 4.4.1 Heterogeneity of shareholdings

There may also be significant differences in the impact of digital technology innovations on the market value of firms with different property rights. Non-state-owned enterprises(NSOEs) are more focused on pursuing economic benefits. Therefore, NSOEs will be more motivated to utilize digital technological innovations, which in turn will result in higher market valuation. Digital technology innovations by SOEs are more likely to be for policy purposes and may not affect the market value of the firm. SOEs face a more favorable external business environment than NSOEs, including easier access to bank loans and more policy incentives, may have less incentive to innovate in digital technology. SOEs do not exist without clear owners, are vulnerable to insider control, weaker levels of corporate governance, leading to inefficient investment, so digital technology innovations that do not play a role in adding value to the firm.

The heterogeneity test is based on dividing the sample into SOEs and NSOEs based on the nature of the actual controller. Column (1) of Table 6 shows that the regression coefficient of *LnDigiInno* for NSOEs is significantly positive at 1% significance level. Column (2) shows that the regression coefficient of *LnDigiInno* for SOEs is significantly positive at 5% significance level. It suggests that the facilitating effect of digital technological innovations on the market value of firms is manifested both in SOEs and NSOEs. But the promotion effect of digital technology innovation on the market value of NSOEs is more significant than SOEs.

**Table 6:** Heterogeneity test.

Variables	(1)	(2)	(3)	(4)
	NSOEs	SOEs	Small	Large
LnDigiInno	0.075***	0.047**	0.024	0.074***
	(4.24)	(2.67)	(1.28)	(4.17)
cons	9.133***	8.968***	18.422***	6.082***
	(13.81)	(13.65)	(16.25)	(15.16)
Control	YES	YES	YES	YES
Fixed	YES	YES	YES	YES
*N*	7806	4051	5542	6247
adj. *R*^2^	0.337	0.468	0.413	0.395

#### 4.4.2 Size heterogeneity

There may be more significant differences in the impact of digital technology innovation on the market value of firms of different sizes. Larger firms with richer data resources and stronger financial strength (Bessen et al., 2019) [50] are more likely to engage in digital technological innovation and obtain more digital technological innovation outputs. It is worth noting that small firms are under-attended by capital, which makes it more important for them to make digital technology innovations to gain access to the market. Once small firms have succeeded in digital technology innovations, more likely to attract a high level of capital attention, which in turn will significantly increase their market value.

A dummy variable for firm size is constructed to verify the difference in the impact of digital technological innovation on firm market value of different sizes. Firm with total assets greater than the industry median is defined as a large, otherwise it is a small or medium-sized firm (Zhang et al., 2021) [13]. Column (3) of Table 6 shows that the *LnDigiInno* regression coefficients for small firms are positive but insignificant. Column (4) shows that the *LnDigiInno* regression coefficients for large firms are significantly positive at the 1% level of significance. It suggests that digital technology innovation contributes to the market value of both small and large firms, but it contributes more significantly to the market value of large firms compared to small firms.

#### 4.4.3 Industry heterogeneity

Referring to the classification method of Lu et al. (2014) [51], classifies listed companies’ industries into labor-intensive(*Labor*), capital-intensive(*Capital*) and technology-intensive(*Tech*). In General, the flow of resources from labor industries to capital and technology industries, represents the direction of high-quality development. More resource-rich industries have stronger incentives to innovate. Column (1) of Table 7 shows that the regression coefficient of *LnDigiInno* for labor firms is significantly positive but not significant. Columns (2) and (3) show that the LnDigiInno regression coefficients for both capital and technology firms are positive at the 1% significance level. The results illustrate that firm digital technology innovation can significantly contribute to the market value of both capital and technology firms.

**Table 7:** Heterogeneity test.

Variables	(1)	(2)	(3)	(4)	(5)
	Labor	Capital	Tech	L-mgtcost	H-mgtcost
LnDigiInno	0.049	0.126***	0.068***	0.055***	0.053***
	(1.56)	(5.48)	(5.09)	(3.40)	(3.04)
cons	5.088***	8.410***	9.668***	6.932***	11.111***
	(6.99)	(12.53)	(16.70)	(13.09)	(14.43)
Control	YES	YES	YES	YES	YES
Fixed	YES	YES	YES	YES	YES
*N*	1120	2439	8477	5883	5847
adj. *R*^2^	0.427	0.381	0.365	0.416	0.386

#### 4.4.4 Internal control cost heterogeneity

The impact of digital technology innovations on firms’ market value may have internal control cost heterogeneity. Digital technology innovations have a more pronounced effect on the market value of firms with low internal control costs. Digital technological innovation is not only conducive to realizing the real-time and transparent activities of the internal management process, R&D process, and production process of the firm, but also reduces the supervision cost of the firm as well as the loss of efficiency caused by the divisional agency problem. It is also conducive to the realization of timely communication and analysis of information among various departments within the firm, optimizing the collaboration and linkage of activities such as feeding, production, transportation and warehousing among divisions, reducing the cost of coordination among departments, and improving the efficiency of management decision-making in the firm. Funds released from low control costs can be used to invest in new technologies, products and market expansion, driving firm innovation and growth, which is reflected in the increased market value of the firm from digital technology innovation. This paper selects the proportion of administrative expenses (Yuan et al., 2021) [5] as a proxy variable for the cost of internal control in firms. The dummy variable *mgtcost* takes the value of 1 when the firm’s management expenses as a share of operating income is below the sample median, and 0 otherwise. Table 7, columns (4)-(5) show that the LnDigiInno regression coefficients are all positive at the 1% significance level, indicating that digital technological innovation contributes to the market value of both high and low internal control cost firms. However, it contributes more to the market value of firms with low internal control costs.

## 5 Discussion

This article focuses on the impact of digital technology innovation on the market value of firms. Similar to previous research on the market value of firms, digital technology innovation can enhance the market value of firms. One possible explanation is that digital technology innovation breaks the boundaries of organizations, expands the value space of firms, and thus enhances the market value of firms. In addition, the digital transformation of firms, production efficiency, and market profitability are three important mechanisms through which digital technology innovation affects the market value of firms.

However, due to the complexity of digital technology innovation itself, there are also some shortcomings. Due to data limitations, this study did not discuss whether there are differences in the impact of digital technology innovation on the market value of enterprises in different regions. This is a potential focus area for future research.

## 6 Conclusions

This paper utilizes patent data to construct a measurement index of firm digital technological innovation. Examined the impact of digital technological innovation on firm market value and its role mechanism and heterogeneity. Research findings:(1) digital technological innovation can enhance firm market value. Patenting digital technology inventions is the main type of innovation that enhances the market value of a firm. (2) Firm digital transformation, production efficiency, and market profitability are important mechanisms. Digital technological innovation enhances firm market value by promoting firm digital transformation. Digital technological innovation enhances firm market value by promoting firm total factor productivity. Digital technological innovation enhances firm market value by improving firm market profitability and internal capital profitability. (3) Heterogeneity analysis shows that the effect of digital technological innovation on the market value of firms is particularly significant among NSOEs, large, capital-intensive, technology-intensive and low internal control costs firms.

The revelations are as follows:

1. digital technology innovation has a contributing effect on firm market value. Therefore, government departments should guide national resources and market resources in favor of digital technological innovation, encourage firms to carry out multifaceted exploration of digital technological innovation, and promote the in-depth fusion of digital technology with the application of supply and demand diversity, enhancing the market value of firms.
2. Accelerate the promotion of firm digital transformation. Digital technology innovation is the key to digital transformation, and firm digital technology innovation has a facilitating effect on firm digital transformation. Therefore, the government can give priority to encouraging firms with knowledge advantages to carry out digital technological innovation and accelerate the integration of digital technology and products and business models. Enterprises should actively study and judge their own digital development needs, technological innovation advantages and the digitalization development trend of the industry, choose suitable application scenarios, promote digital technological innovation, accelerate digital transformation, make the results of digital technological innovation contribute to the market value of firms.
3. It emphasizes the role of production efficiency and market profitability. Firms should pay attention to the improvement of production efficiency, accelerate the application of production line digitization, and improve the construction of firm digital system. Building a digital network management platform, quickly and accurately capture the market demand, realize digital precision marketing, and effectively enhance the firm market value.
4. There are differences in the impact of digital technology innovation on firms’ market value across firms. The government needs to take into account the heterogeneity of firms and industries when formulating policies, apply policies with precision. Small and labor-intensive firms have problems such as insufficient R&D funding, financing constraints, a shortage of talent for digital technology innovation. So government should improve its financial subsidy policy to provide safeguards for firms’ digital technology innovation.

## Supporting information

S1 Data (XLSX)

## References

[1] Tian XJ, Li R. Digital technology empowers the transformation and development of real economy-an analytical framework based on Schumpeter’s endogenous growth theory. Management World. 2022;38(05):56–74.

[2] Qi ID, Du B, Wen W. Digital strategic change of state-owned enterprises: mission embeddedness and mode selection—a case study based on the digitalization typical practices of three central enterprises. Management World. 2021; (11), 137–158+10.

[3] Svahn F L Mathiassen R Lindgren. Embracing Digital Innovation in Incumbent Firms: How Volvo Cars Managed Competing Concerns. MIS Quarterly. 2017;41(1),239–253.

[4] Acemoglu D P Restrepo. The Race between Man and Machine: Implications of Technology for Growth, Factor Shares, and Employment. American Economic Review. 2018;108(6),1488–1542.

[5] Yuan C, Xiao TS, Geng CX, Sheng Y. Digital transformation and corporate division of labor: specialization or vertical integration. China Industrial Economics. 2021;(09), 137–155.

[6] Ciarli T, Kenney M, Massini S, Piscitello L. Digital Technologies, Innovation, and Skills:Emerging Trajectories and Challenges. Research Policy. 2021; 50(7),104289.

[7] Bharadwaj A S, Bharadwaj S G, Konsynski B R. Information Technology Effects on Firm Performance as Measured by Tobin’s q. Management Science.1999;45 (7), 1008–1024.

[8] Bajari P, Chernozhukov V, Hortaçsu A, Suzuki J. The Impact of Big Data on Firm Performance: An Empirical Investigation. AEA Papers and Proceedings. 2019;109, 33–37.

[9] Cathles A, Nayyar G, Rückert D. Digital Technologies and Firm Performance: Evidence from Europe. EIB Working Papers. 2020.

[10] Liu Y, Dong J, Mei L, Shen R. Digital Innovation and Performance of Manufacturing Firms: An Affordance Perspective. Technovation. 2023;119, 102458.

[11] Firk S Y Gehrke, Hanelt A, Wolff M. Top Management Team Characteristics and Digital Innovation: Exploring Digital Knowledge and TMT interfaces. Long Range Planning. 2021;55 (3),102166.

[12] Teece DJ. Profiting from Innovation in the Digital Economy: Enabling Technologies, Standards, and Licensing Models in the Wireless World. Research Policy. 2018;47(8),1367–1387. doi: 10.1016/j.respol.2017.01.015

[13] Zhang YQ, Lu Y, Li LY. The impact of big data applications on the market value of Chinese firms—Evidence from textual analysis of annual reports of Chinese listed companies. Economic Research. 2021; (12), 42–59.

[14] Wu F, Hu HZ, Lin HY, Ren XY. Corporate digital transformation and capital market performance—Empirical evidence from stock liquidity. Management World. 2021;(07), 130–144+10.

[15] Zhang ME, Li HP, Ren TF. A Study of Strategic Patenting Behavior and Interaction in Digital Innovation. Research in Science, 2022;(03), 545–554.35113718

[16] Kohli R, Melville N P. “Digital Innovation: A Review and Synthesis”, Information Systems Journal. 2019;29 (1), 200–223.

[17] Tumbas S, Berente N, Brocke J. “Digital Innovation and Institutional Entrepreneurship: Chief Digital Officer Perspectives of their Emerging Role”, Journal of Information Technology. 2018;33 (3), 188–202.

[18] Yoo Y, Boland, Lyytinen K, Majchrzak A. Organizing for Innovation in the Digitized World. Organization Science. 2012;23(5),1398–1408.

[19] Qu YY. Organizational foundations of digital innovation and heterogeneity in China. Management World. 2022; (10), 158–174.

[20] Tee R, Gawer A. Industry Architecture as a Determinant of Successful Platform Strategies: A Case Study of the I-Mode Mobile Internet Service. European Management Review. 2009;6 (4),217–232.

[21] Balci G. Digitalization in Container Shipping: Do Perception and Satisfaction Regarding Digital Products in a Non-technology Industry Affect Overall Customer Loyalty?. Technological Forecasting and Social Change. 2021;172,121016.

[22] Yoo Y, Henfridsson O, Lyytinen. Research Commentary-the New Organizing Logic of Digital Innovation: An Agenda for Information Systems Research. Information Systems Research. 2010;21 (4),724–735.

[23] Lyytinen K, Yoo Y, Boland Jr R J. Digital Product Innovation Within Four Classes of Innovation Networks. Information Systems Journal. 2016;26 (1),47–75. doi: 10.1111/isj.12093

[24] Wei LL, Hou YQ. A study on the role of digital economy in influencing the green development of Chinese cities. Research on Quantitative and Technical Economics. 2022; (08), 60–79.

[25] Huang QH, Yu YZ, Zhang SL. Internet development and manufacturing productivity enhancement: intrinsic mechanism and Chinese experience. China Industrial Economics. 2019; (08), 5–23.

[26] Xiao JH. Enterprise cross-system digital transformation and management adaptive change. Reform. 2020; (04), 37–49.

[27] Brynjolfsson E, Hitt L M, Kim H H. Strength in Numbers: How does Data-Driven DecisionMaking Affect Firm Performance? Working paper. 2011.

[28] Nambisan S, Lyytinen K, Majchrzak A, Song M. Digital Innovation Management: Reinventing Innovation Management Research in a Digital World. MIS Quarterly. 2017;41 (1),223–238.

[29] Bharadwaj A Sawy O A, Pavlou P. A., Venkatraman N. MIS Quarterly. 2013;37(2),471–482. Digital Business Strategy: Toward a Next Generation of Insights.

[30] Brynjolfsson E Mitchell T. What Can Machine Learning Do? Workforce Implications. Science. 2017;358 (6370), 1530–1534. 29269459 10.1126/science.aap8062

[31] Babina T Fedyk A, He A X, & Hodson J. Artificial Intelligence, Firm Growth, and Industry Concentration. Working Paper.2021.

[32] Grover V, Kohli R. Cocreating IT Value: New Capabilities and Metrics for Multifirm Environments. MIS Quarterly. 2012;36 (1),225–232.

[33] Henfridsson O, Nandhakumar J, Scarbrough H, Panourgias N. Recombinati-on in the OpenEnded Value Landscape of Digital Innovation. Information and Organization. 2018;28 (2),89–100.

[34] Yu J, Meng QS, Zhang Y, Zhang X, Chen F. Digital innovation:Exploration and insights of new perspectives in innovation research. Research in Science. 2017; (07), 1103–1111.

[35] Mendling J, Pentland B T, Recker J. Building a Complementary Agenda for Business Process Management and Digital Innovation. European Journal of Information Systems. 2020;29(3),208–219.

[36] Chen DQ, Hu Q. Corporate governance research in the era of digital economy: paradigm innovation and practice frontiers. Management World. 2022; (06), 213–240.

[37] Li MY, Yan TH. Venture capital, technological innovation and firm performance: influence mechanism and its empirical test. Research Management,2020; 41(07), 70–78.

[38] Li F. The Digital Transformation of Business Models in the Creative Industries: A Holistic Framework and Emerging Trends. Technovation,2020; 92,102012.

[39] Li DH, Chen YR, Zhou PL. Cracking the cross-border network governance path of disruptive technological innovation—a case study based on Baidu Apollo autonomous driving open platform. Management World, 2021;(4), 130–159.

[40] Liu Y, Dong JY, Wei J. Digital innovation management: theoretical framework and future research. Management World.2020;(07), 198–217+219.

[41] Guellec D, Paunov C. Digital Innovation and the Distribution of Income. Working Paper. 2017.

[42] Tao F, Zhu P, Qiu CZ, Wang X R. A study on the impact of digital technology innovation on firms’ market value. Research on Quantitative and Technical Economics.2023; (05), 68–91.

[43] Luo K, Zuo XT. An unbalanced research on the impact of patent jungle on firm market value. Research Management,2023;44(10),168–180.

[44] Huang B, Li HT, Liu JQ, Lei JH. Digital technology innovation and high-quality development of Chinese firms—Evidence from firms’ digital patents. Economic Research. 2023; (03), 97–115.

[45] Wu CP, Tang G. Intellectual Property Protection Enforcement Effort, Technological Innovation and Firm Performance-Evidence from Chinese Listed Companies. Economic Research. 2016; (11), 125–139.

[46] Jiang T. Mediating effects and moderating effects in causal inference. China Industrial Economics. 2022; (05), 100–120.

[47] Li JQ, Zhao XL. Labor protection and corporate innovation—an empirical study based on the Labor Contract Law. Economics (Quarterly). 2021;(01), 121–142.

[48] Liu Q, Lu Y. “Firm Investment and Exporting: Evidence from China’s Value-added Tax Reform”, Journal of International Economics. 2015;97 (2),392–403.

[49] Liu Q, Qiu LD Intermediate Input Imports and Innovations: Evidence from Chinese Firms’ Patent Filings. Journal of International Economics. 2016;103,166–183.

[50] Bessen J, Righi C. Shocking Technology: What Happens When Firms Make Large IT Investments?. Law and Economics Research Paper No. 19–6, Boston University School of Law. 2019.

[51] Lu T, Dang Y. Corporate governance and technological innovation: a comparison by industry. Economic Research (06). 2014;115–128.

